# Supplementary material for: Plasmodium falciparum 7G8 challenge provides conservative prediction of efficacy of PfNF54-based PfSPZ Vaccine in Africa
Source: Nat Commun. 2022 Jun 13;13:3390. doi: 10.1038/s41467-022-30882-8 (PMC9189790; doi:10.1038/s41467-022-30882-8)
Supplement: Supplementary file 1 — Supplementary Information [file 41467_2022_30882_MOESM1_ESM.pdf]

Supplemental Information for

Plasmodium falciparum 7G8 challenge provides conservative prediction of efficacy of PfNF54-based PfSPZ Vaccine in Africa

Joana C. Silva, Ankit Dwivedi, Kara A. Moser, Mahamadou S. Sissoko, Judith E. Epstein, Sara A. Healy, Kirsten E. Lyke, Benjamin Mordmüller, Peter G. Kremsner, Patrick E. Duffy, Tooba Murshedkar, B. Kim Lee Sim, Thomas L. Richie, Stephen L. Hoffman

To determine the cause of the multi-modal distribution of genetic distances to NF54, observed for all three African regions (Figures 1, 2), we investigated the relationship between genetic distance and three sample characteristics, namely country of origin, complexity of infection (as measured by F_{WS}[1,2], and data missingness (percent of positions called as missing). F_{WS} varied between 1 (single clone) to 0 (multiple clones at similar proportions in the sample). Monoclonal samples characterized by F_{WS}>0.95. We used Principal Components Analysis (PCA) to investigate these relationships.

F_{WS} was calculated for each sample using all called quality-filtered SNPs in the core nuclear genome, per country, with the R package moimix (https://github.com/bahlolab/moimix). PCA plots were created in R using the gdsfmt and SNPRelate packages[3]. PCAs were based on the quality-filtered, bi-allelic positions, in the core region of the 14 nuclear chromosomes, used to determine nonsynonymous genetic distances in epitope regions (Figure 2B), i.e., all non-synonymous sites in predicted epitopes. SNPs in allelic association were removed by calculating linkage disequilibrium (LD) with a sliding window of 500Kb and pruning SNPs with LD>0.2.

Analyses were conducted separately for each of three main African regions, to eliminate the confounding effect from the association between genetic distance to NF54 and geography (Figure 1). However, the conclusions were similar across regions. Taking the example of West Africa (Mali, Burkina Faso and Guinea; Supplemental Figure S1), samples with smaller distances to NF54 (in red, in Figure S1.A, clustered around PC coordinates 0,0) were not associated with country, but were associated with higher complexity of infection (i.e, low F_{WS}; Figure S1.B), and higher proportions of missing data (high missingness, Figure S1.C). The connection between complexity of infection and missing data could be explained as follows.

Samples with lowest values of F_{WS} were composed of two or more clones, in balanced proportions. These samples were more likely to have bi-allelic positions in which neither allele was represented in >70% of reads. Positions with this characteristic were converted to missing data as part of the SNP calling algorithm (Methods). As a result, polyclonal samples with balanced clone composition had a higher proportion of missing positions, and these missing positions tended to be variable. This artificially reduced genetic distance to NF54. In fact, data missingness was highly correlated with genetic distance to NF54, with R^2 ~92% (Supplemental Figure S1.D).

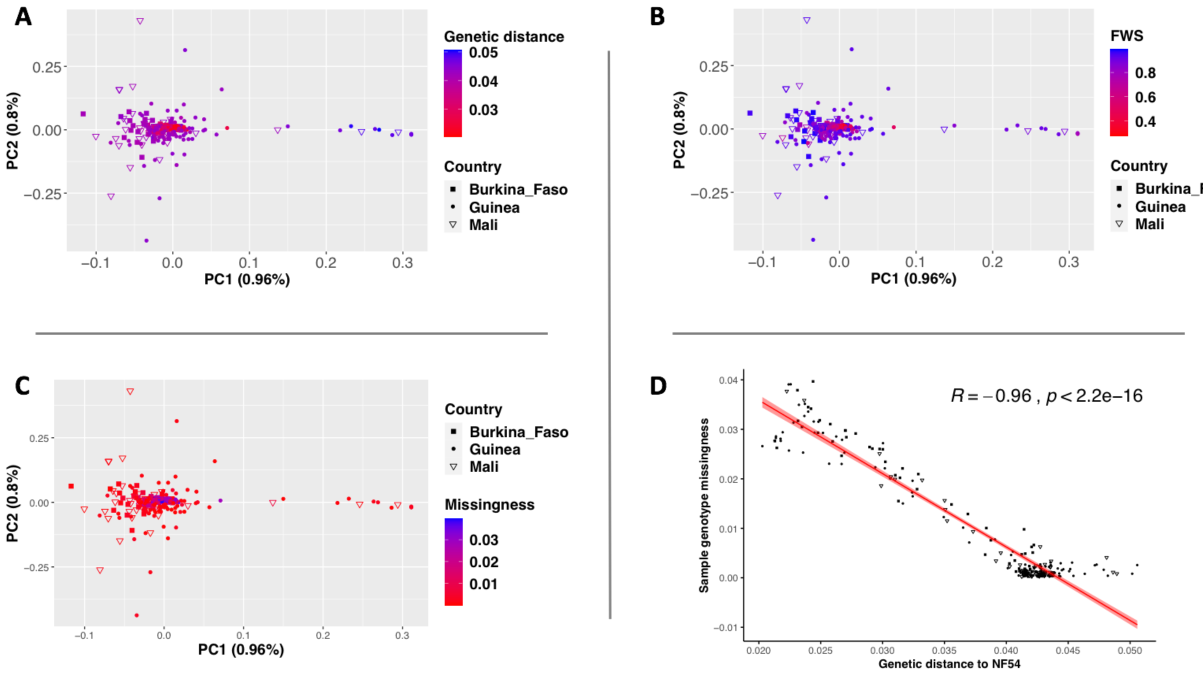

Supplemental Figure S1. Relationship between genetic distance to PfNF54 and characteristics of samples from West Africa. **A.** Each sample in the PCA plot is marked by geographic region (shape: Burkina Faso, filled square; Guinea, filled dot; Mali, open triangle), with color reflecting genetic distance to PfNF54 (from lowest, in red, to highest in blue). Samples with lowest genetic distance to NF54 clustered around coordinates 0,0 (PC1, PC2). Samples did not cluster by country, suggesting that the *P. falciparum* population in this region of West Africa is fairly panmictic, as seen previously[4]. **B.** Samples clustered around coordinates 0,0 (PC1, PC2) also had lowest F_{WS} (bright red), meaning that they had higher complexity of infection. **C.** Samples clustered around coordinates 0,0 (PC1, PC2) had the most missing data (blue). **D.** The correlation between missingness and genetic distance was investigated using Pearson's correlation coefficient, R. The proportion of the variation in the data that is explained by the relationship between the variables "Genotype missingness" and "Genetic distance to PfNF54" (estimated by R^2) is ~92%.

Results were similar for samples from central Africa (Supplemental Figure S2). Namely, data missingness explained ~94% of the variation in genetic distance to NF54 (Supplemental Figure S2.D). As expected, and unlike for West Africa, the PCA separated the samples from central Africa (Cameroon) from those from South central Africa (Democratic Republic of Congo, DRA) (Supplemental Figure S2.A), known to have different genetic composition[4].

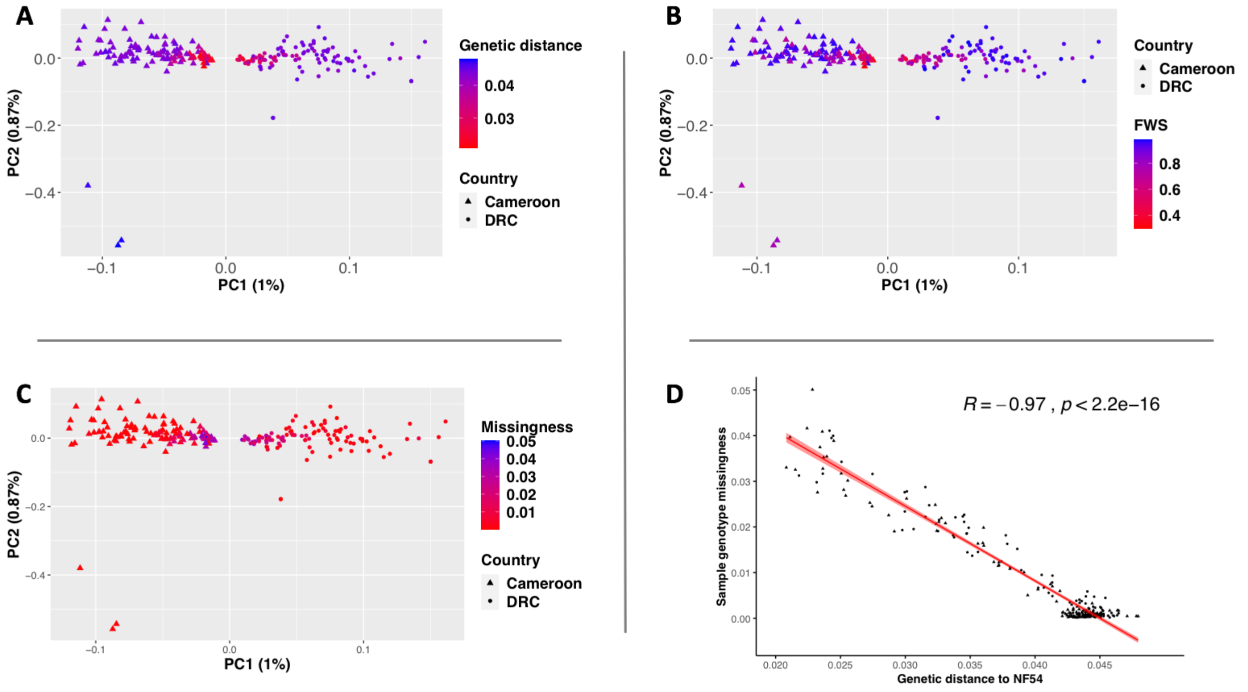

Supplemental Figure S2. Relationship between genetic distance to PfNF54 and characteristics of samples from Central Africa. Legend as in Supplemental Figure S1. **A.** The first PC separated Central Africa (Cameroon; triangles) from South central Africa (Democratic Republic of Congo, DRC; circles). **B, C.** Genetic distance to PfNF54 was positively associated with F_{WS} and inversely associated with data missingness. **D.** As observed for West African samples, among Central African samples data missingness explained nearly all variation in the genetic distance data to Pf NF54 (correlation between variables estimated using Pearson's correlation coefficient, *R*. R^2~94%. Not adjusted for multiple comparisons).

Finally, the samples from East Africa revealed a similar pattern. In this case, the SNP-based PCA separated samples from East Africa (Kenya and Tanzania) from those from Southeast Africa

(Malawi and Madagascar) (Supplemental Figure S3). Conclusions regarding variation in genetic distance to NF54 were similar to those above.

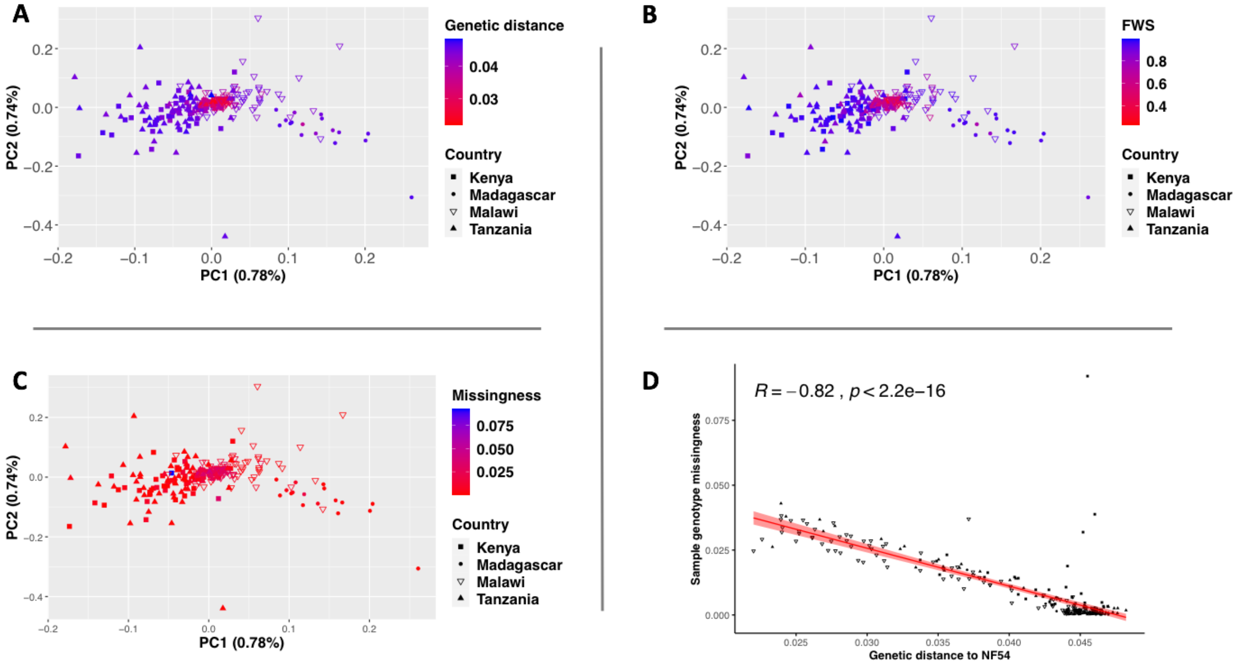

Supplemental Figure S3. Relationship between genetic distance to PfNF54 and characteristics of samples from East Africa. Legend as in Supplemental Figure S1. **A.** The first PC separated East Africa (Kenya and Tanzania; full triangles and squares) from Southeast Africa (Malawi and Madagascar; full circles and empty triangles). **B, C.** Genetic distance to PfNF54 was positively associated with F_{WS} and inversely associated with data missingness. **D.** Data missingness explained most of the variation in the data (correlation between variables estimated using Pearson's correlation coefficient, *R; R^2*~67%. Not adjusted for multiple comparisons).

In addition to calculating *p* distances between PfNF54 and each isolate, limiting analysis only to sites genotyped in Pf7G8, we also calculated identity by state (IBS), focusing now on similarities rather than differences, and using all sites present in each sample or strain in the pairwise comparison (Supplemental Figure S4). As expected for the complement of *p* distance, IBS with PfNF54 decreases slightly from West (median IBS ~96.55%) to Central (median IBS ~96.45%) and to East Africa (median IBS ~96.38%), and is much lower with samples from South America (median ~95.60%), including Pf7G8 (IBS 95.64%). IBS in Africa differs very slightly from (1-*p*) because calculations were not limited to sites genotyped in Pf7G8.

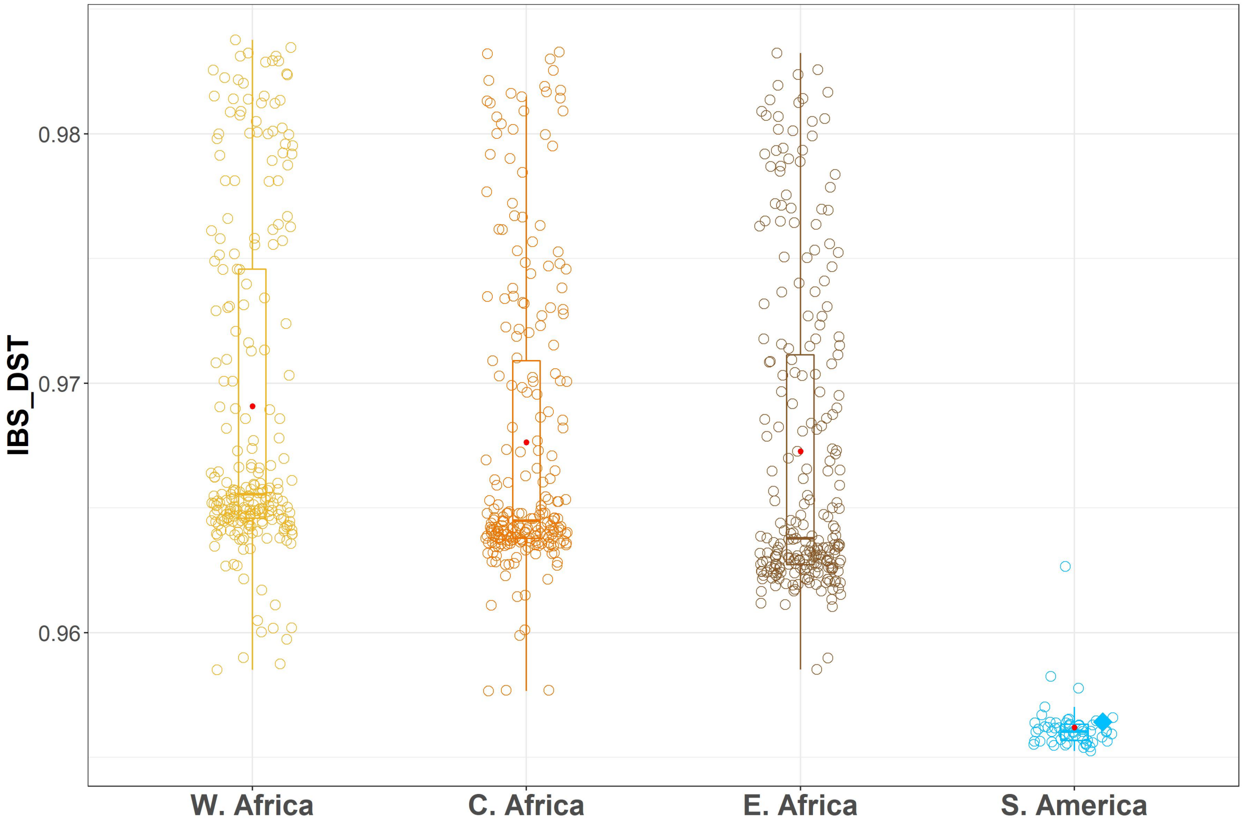

Supplemental Figure S4. Identity-by-state (IBS) between PfNF54 and samples from Africa and South America. IBS between PfNF54 and samples from West Africa (gold; Mali, Guinea and Burkina Faso; n=234), Central Africa (orange; Cameroon and the Democratic Republic of the Congo; n=229), East Africa (brown; Kenya, Tanzania, Malawi and Madagascar; n=241) and South America (blue; Brazil, n=23; French Guiana, n=34). In each case, box plots represent the distribution, with mean (red dot), median (horizontal line) and second and third quartiles (within box) shown. Pf7G8 is shown among South American samples (blue diamond).

References

1. Manske, M., *et al.* Analysis of Plasmodium falciparum diversity in natural infections by deep sequencing. *Nature* **487**, 375-379 (2012).
2. Chan, E.R., *et al.* Whole genome sequencing of field isolates provides robust characterization of genetic diversity in Plasmodium vivax. *PLoS Negl Trop Dis* **6**, e1811 (2012).
3. Zheng, X., *et al.* A high-performance computing toolset for relatedness and principal component analysis of SNP data. *Bioinformatics* **28**, 3326-3328 (2012).
4. Amambua-Ngwa, A., *et al.* Major subpopulations of Plasmodium falciparum in sub-Saharan Africa. *Science* **365**, 813-816 (2019).
