# Supplementary material for: Plasmodium falciparum 7G8 challenge provides conservative prediction of efficacy of PfNF54-based PfSPZ Vaccine in Africa
Source: Nat Commun. 2022 Jun 13;13:3390. doi: 10.1038/s41467-022-30882-8 (PMC9189790; doi:10.1038/s41467-022-30882-8)
Supplement: Supplementary file 3 — Description of Additional Supplementary Files [file 41467_2022_30882_MOESM3_ESM.pdf]

Description of Additional Supplementary Files

File Name: Supplementary Data 1
Description: Samples used for analyses in the manuscript "Genome, proteome, and immunome data explain why 6 month controlled human malaria infection with sporozoites of the Pf7G8 clone of Plasmodium falciparum is a rigorous predictor of the efficacy of the PfNF54-based PfSPZ Vaccine in Africa"
