# Supplementary material for: Plasmodium falciparum 7G8 challenge provides conservative prediction of efficacy of PfNF54-based PfSPZ Vaccine in Africa
Source: Nat Commun. 2022 Jun 13;13:3390. doi: 10.1038/s41467-022-30882-8 (PMC9189790; doi:10.1038/s41467-022-30882-8)
Supplement: Supplementary file 5 — Reporting Summary [file 41467_2022_30882_MOESM5_ESM.pdf]

nature research

Corresponding author(s): Stephen L. Hoffman

Last updated by author(s): May 9, 2022

Reporting Summary

Nature Research wishes to improve the reproducibility of the work that we publish. This form provides structure for consistency and transparency in reporting. For further information on Nature Research policies, see our Editorial Policies and the Editorial Policy Checklist.

Statistics

For all statistical analyses, confirm that the following items are present in the figure legend, table legend, main text, or Methods section.

n/a	Confirmed	
☐	☒	The exact sample size (*n*) for each experimental group/condition, given as a discrete number and unit of measurement
☒	☐	A statement on whether measurements were taken from distinct samples or whether the same sample was measured repeatedly
☐	☒	The statistical test(s) used AND whether they are one- or two-sided<br>*Only common tests should be described solely by name; describe more complex techniques in the Methods section.*
☒	☐	A description of all covariates tested
☒	☐	A description of any assumptions or corrections, such as tests of normality and adjustment for multiple comparisons
☐	☒	A full description of the statistical parameters including central tendency (e.g. means) or other basic estimates (e.g. regression coefficient) AND variation (e.g. standard deviation) or associated estimates of uncertainty (e.g. confidence intervals)
☐	☒	For null hypothesis testing, the test statistic (e.g. *F*, *t*, *r*) with confidence intervals, effect sizes, degrees of freedom and *P* value noted<br>*Give P values as exact values whenever suitable.*
☒	☐	For Bayesian analysis, information on the choice of priors and Markov chain Monte Carlo settings
☒	☐	For hierarchical and complex designs, identification of the appropriate level for tests and full reporting of outcomes
☐	☒	Estimates of effect sizes (e.g. Cohen's *d*, Pearson's *r*), indicating how they were calculated

Our web collection on statistics for biologists contains articles on many of the points above.

Software and code

Policy information about availability of computer code

Data collection	No software was used for data collection.
Data analysis	The following software was used in the analysis of the data, all of which are cited in the manuscript: bowtie2 (v2.2.4), Haplotype Caller (v4.0), snpEFF (v4.3t), NetMHCpan (v4.0a), packages in R (versions v4.0.0 and later) for PCA analysis and figure plotting, PLINK v1.90.

For manuscripts utilizing custom algorithms or software that are central to the research but not yet described in published literature, software must be made available to editors and reviewers. We strongly encourage code deposition in a community repository (e.g. GitHub). See the Nature Research guidelines for submitting code & software for further information.

Data

Policy information about availability of data

All manuscripts must include a data availability statement. This statement should provide the following information, where applicable:

- Accession codes, unique identifiers, or web links for publicly available datasets
- A list of figures that have associated raw data
- A description of any restrictions on data availability

All whole genome sequence datasets used in this study are available for download through the NCBI's Sequence Read Archive (SRA) database. Accession IDs are available in Supplemental Table 1.

Field-specific reporting

Please select the one below that is the best fit for your research. If you are not sure, read the appropriate sections before making your selection.

☐ Life sciences ☐ Behavioural & social sciences ☒ Ecological, evolutionary & environmental sciences

For a reference copy of the document with all sections, see nature.com/documents/nr-reporting-summary-flat.pdf

Ecological, evolutionary & environmental sciences study design

All studies must disclose on these points even when the disclosure is negative.

Study description	Whole genome sequence data from Plasmodium falciparum isolates (Africa, n=704) were compared with the genome sequence of the P. falciparum strain used in PfSPZ Vaccine, named PfNF54. Those differences were quantified for three data partitions: whole genome, non-synonymous sites and epitope-coding sites. The results were compared with genetic distances obtained similarly for the P. falciparum strain used in heterologous CHMI named Pf7G8. All isolates were treated as independent from each other.
Research sample	The source of each dataset is listed in Supplemental Table 1. Datasets were selected from malaria-endemic countries in West, Central and East Africa, to obtain a comprehensive representation of the genetic diversity across the continent. Within each country, samples were from a single study and were chosen randomly
Sampling strategy	The sample size was selected to represent an adequate balance between comprehensiveness and analysis time (ie., as many as reasonably required). East and West African countries harbor the P. falciparum populations that are most genetically distinct from each other in the continent (with Central Africa P. falciparum populations being roughly intermediate between those from E and W Africa), hence encompassing the range of observable P. falciparum genetic variation in Africa. A roughly similar number of samples was used from each of the three geographic regions. A sample size of ~50 isolates per geographic region (here represented by each country) is generally considered as a reasonable representation of genome-wide variation in P. falciparum populations.
Data collection	Co-author AD downloaded all data from public repositories.
Timing and spatial scale	For each of the countries for which whole genome sequencing datasets were available in SRA, samples were selected from a single study (as far as we cal tell, each from a single town or district), with all collection times listed as falling in the interval 2007-2016, to minimize possibly effects of parasite population evolution.
Data exclusions	Only bi-allelic sites were used, to avoid rapidly evolving sites. For heterozygous sites, the major allele was called if supported by >70% of reads, and otherwise coded as missing. The following stringent hard filter was also applied, to eliminate any low quality SNP positions: DP < 12 || QUAL < 50 || FS > 14.5 || MQ < 20. Finally, variants were filtered out if present in fewer than three samples or when missing genotype values were present in 10% or more of all samples.
Reproducibility	Reproducibility was ensured by using whole genome sequencing data, and often all datasets available. Robustness of the results was achieved by using hundreds of datasets from each geographic region.
Randomization	Each geographic region is inherently represented by samples collected within it and therefore there are no issues related to randomization of samples across sites.
Blinding	For each country, if the total number of samples/datasets available exceeded the number desired, datasets were randomly picked, without a priori knowledge of genetic distance to other samples or to the strain in the vaccine.

Did the study involve field work? ☐ Yes ☒ No

Reporting for specific materials, systems and methods

We require information from authors about some types of materials, experimental systems and methods used in many studies. Here, indicate whether each material, system or method listed is relevant to your study. If you are not sure if a list item applies to your research, read the appropriate section before selecting a response.

Materials & experimental systems

n/a	Involved in the study
☒	☐ Antibodies
☒	☐ Eukaryotic cell lines
☒	☐ Palaeontology and archaeology
☒	☐ Animals and other organisms
☒	☐ Human research participants
☒	☐ Clinical data
☒	☐ Dual use research of concern

Methods

n/a	Involved in the study
☒	☐ ChIP-seq
☒	☐ Flow cytometry
☒	☐ MRI-based neuroimaging
